# The anti-COVID-19 drug Favipiravir: Degradation, Method development, Validation, NMR/LC–MS characterization, and *In-vitro* safety evaluation

**DOI:** 10.1007/s11696-022-02327-5

**Published:** 2022-07-04

**Authors:** Inas A. Abdallah, Mohammed F. El-Behairy, Rasha M. Ahmed, Marwa A. A. Fayed

**Affiliations:** 1 Department of Analytical Chemistry, Faculty of Pharmacy, University of Sadat City, Sadat City, 32897 Egypt; 2 Department of Organic and Medicinal Chemistry, Faculty of Pharmacy, University of Sadat City, Sadat City, 32897 Egypt; 3 Department of Pharmaceutical Chemistry, Faculty of Pharmacy, Misr International University, Cairo, Egypt; 4 Department of Pharmacognosy, Faculty of Pharmacy, University of Sadat City, Sadat City, 32897 Egypt

**Keywords:** Favipiravir, COVID-19, Safety, Degradation, Oxidative, Alkaline, SRB assay, Cytotoxicity, NHSF cells

## Abstract

It is critical to characterize the degradation products of therapeutic drugs to determine their safety as these degradation products may possess fatal effects on the human physiological system. Favipiravir (FVP), a novel anti-Covid-19 drug, that is recently used all over the world with a great impact on humanity was our target to explore more about its toxicity, the margins of its safety, and its degradants in different degradation conditions. The goal of this study is to identify, characterize, and confirm the structures of FVP oxidative and alkaline breakdown products, as well as to assess their safety utilizing *in-vitro* SRB cytotoxicity assay on normal human skin fibroblasts (NHSF) cell lines. After oxidative and alkaline degradation of FVP, one degradation product was produced in each condition which was isolated from FVP using flash chromatography, characterized by ^1^HNMR and LC–MS/MS techniques. A reversed-phase Thermo Fischer Hypersil C_18_ column (4.6 × 150 mm, 5 m) was used to achieve HPLC chromatographic separation. Acetonitrile-5 mM potassium dihydrogen phosphate (pH 2.5) (50:50, v/v) was employed as the mobile phase, with a flow rate of 1 mL/min. At 332 nm, the column effluent was measured. Over the concentration range of 0.5–100 µg/mL, the calibration curve was linear. The intra-day and inter-day relative standard deviations were less than 2%, and good percentage recoveries were obtained that fulfilled the acceptance criteria of the International Conference on Harmonization (ICH) recommendations. The Plackett–Burman design was used to assess the robustness. Each degradant was isolated single using Flash chromatography and methylene chloride: methanol gradient mobile phase. The chemical structures of the degradation products have been confirmed and compared to the intact FVP using ^1^H-NMR, and Mass spectroscopy. A postulated mechanism of the degradation process has been depicted and the degradants fragmentation pattern has been portrayed. In addition, the *in vitro* SRB cytotoxicity assay to evaluate the safety profile of FVP and the degradation end products showed their high safety margin in both conditions with IC_50_ ˃100 µg/ml with no signs of toxicity upon examination of the treated NHSF cells under the optical microscope

## Introduction

Favipiravir (FVP) is a novel antiviral drug used as a medication for a pandemic influenza infection in Japan (Furuta et al. 2017). The activation process of a prodrug of FVP was performed by intracellular phosphorylation to the actively favipiravir ribofuranosyl-5B-triphosphate. FVP targeted the prevention of viral replication through selective suppression of RNA-dependent RNA polymerase (RdRp) of influenza viruses (Bai et al. 2016; Tanaka et al. 2017). After numerous clinical trials to evaluate the suitability for potential repurposing of FVR in Covid 19, it was recommended to use FVP as a medication for the treatment of SARS-CoV2 because the selectivity index against SARS-CoV2 was 6.46 (Agrawal et al. 2020; Elfiky, 2021; Ivashchenko et al. 2021).

Degradation of the active ingredients and verification of their acceptable safety limits according to ICH guidelines, are of great importance (CPMP, 2007; ICH Q1A(R2), 2003). Thus, the stability of drug substances and pharmaceutical dosage forms are evaluated and tested under drastic conditions by studying the effect of different factors like heat, pH of the medium, and light to understand the behavior of drug molecules (Blessy et al. 2014). Identification of the degradation products followed by isolation and characterization steps are helpful to predict the degradation mechanism of the drug molecule subjected to various conditions such as hydrolysis, oxidation, and photolysis (Udutha et al. 2021). Furthermore, the toxicity of isolated degradation products can be evaluated by performing *in vivo* and *in vitro* toxicity studies (M. A. Mohd Shafiee, 2021; Wang et al. 2002).

After a literature survey, It was found that few analytical methods were reported for estimation of FVP in different matrices using different techniques; spectrophotometric (BULDUK, 2021), spectrofluorimetric (Mikhail et al. 2021) chromatographic (Abdallah et al. 2022; BULDUK, 2021; I. A. Abdallah, 2022; Kaddah et al. 2021; Mikhail et al. 2021; M. I. Morsy et al. 2021; Mosaad I. Morsy et al. 2021), and voltametric methods (Mehmandoust et al. 2021). Although few stability-indicating HPLC methods were previously reported, no comprehensive stability-indicating method with isolation and characterization of the degradation products has been published yet for the quantification of FVP. Moreover, the studied drug was subjected in the reported methods to forced degradation study using different conditions resulting in different degradation pathway.(Gökce, 2021; Lingabathula, 2021; Marzouk et al. 2022; Mosaad I. Morsy et al. 2021).

In the presented work, an attempt has been made to study the oxidative and alkaline degradation products of FVP as per ICH guidelines (ICH Q1A(R2), 2003), isolation, and characterization using flash chromatography and HPLC–MS respectively. Further, based on the MS and ^1^H-NMR of the obtained degradation products, chemical structures have been elucidated and the degradation mechanism has been predicted. This represents the first study to isolate and characterize the structures of FVP forced degradation products and their safety potential.

## Materials and methods

### Chemicals and reagents

FVP with a purity of 99.7% was graciously provided by Liptis Pharmaceutical Industries (Cairo, Egypt). Sigma Aldrich provided HPLC-grade methanol, acetonitrile, hydrochloric acid (HCl), sodium hydroxide (NaOH), deuterated chloroform (CDCl_3_), and deuterated DMSO (DMSO-*d*_*6*_) (St. Louis, MO, USA). J.T. Baker Chemical Co. provided potassium dihydrogen phosphate, phosphoric acid, sodium hydroxide, and hydrogen peroxide (30% w/v) (Phillipsburg, NJ, USA). A MilliQ plus water system filtered distilled water (Millipore; Billerica, MA, USA).

## Instrumentation

The DionexUltiMateTM 3000 HPLC (Thermo Scientific, Dionex, Sunnyvale, CA, USA) was used for chromatographic separations. A WPS-3000TSL autosampler, an LPG-3400SD quaternary pump, a VWD-3000 variable wavelength detector, and a TCC-3000SD column thermostat comprise the instrument. Chromeleon 7 software was used to process and collect data.

Isolation of oxidative and alkaline degradation products of FVP was carried out on Flash chromatography apparatus (puriFlash XS 520 Plus) (Interchim, France).

Analyst 1.6.3 software was used to collect the mass spectrometric data. For MS scans to identify FVP and its degradation products (FDP1 & FDP2), the following operating source conditions were optimized: 20 psi curtain gas, 5500 V ion spray voltage, 500 °C temperature, 30 V declustering potential, 20 psi ion source gas 1 (GS 1), 20 psi ion source gas 2 (GS 2) The primary nebulizer gas was air, while the secondary gas was nitrogen.

The ^1^H-NMR analysis was carried out on a 400 MHz NMR spectrometer (Bruker, Fällanden, Switzerland) with DMSO-*d*_*6*_ as a solvent. The chemical shifts are expressed as % of TMS (= 0.00 ppm) as the internal standard.

BMG LABTECH- FLUOstar Omega (Ortenberg, Germany) microplate reader was used to measure the absorbance at 540 nm in the cytotoxicity experiment.

## HPLC chromatographic conditions

A Thermo Fischer Hypersil C_18_ column was used to achieve chromatographic separations (150 × 4.6 mm, 5 mm). At a flow rate of 1 mL/min, the mobile phase was made up of (A) acetonitrile and (B) 5 mM potassium dihydrogen phosphate (pH = 2.5) (A: 50, B: 50, v/v). FVP and its degradation products were determined using a 323 nm detection wavelength and a 10 µL injection volume. All the chromatograms were taken at 30 °C.

## Forced degradation study

The forced deterioration testing was conducted in methanol at a concentration of 1 mg/mL. The drug was alkaline hydrolyzed by placing it in a boiling water bath kept at 100 °C for 2 h after being treated with 1 M sodium hydroxide. The drug was oxidatively degraded by immersing it in a boiling water bath at 100 °C for 2 h after being treated with 10% hydrogen peroxide.

## Preparation of samples for chromatographic analysis

To produce the required assay values of 100 µg/mL, the samples from the alkaline and oxidative hydrolysis stress tests were neutralized and diluted with methanol. Before HPLC analysis, the final sample solutions were filtered through a 0.45 µm membrane. To avoid degradation, all sample solutions were kept in a refrigerator at 4 °C.

## Method validation

As indicated in the ICH recommendations Q2 (R1) (ICH Q1A(R2), 2003), the suggested chromatographic technique was validated in terms of selectivity, the limit of detection (LOD), the limit of quantification (LOQ), linearity, accuracy, precision, and robustness (R1).

## Isolation of degradation products

A flash chromatography apparatus (puriFlash XS 520 Plus) was used for the isolation of the degradation products (FDP) of FVP using a normal phase silica gel column (12 g 30 mm) and methylene chloride/methanol gradient. The FDP mixture is composed of one degradation product (FDP1) and the drug FVP from the oxidative degradation and another mixture from the alkaline degradation consists of one degradation product (FDP 2) and FVP. Both mixtures were introduced to flash chromatography separately with a mobile phase composed of methylene chloride/methanol gradient. The flow rate was set at 15 mL/min and fractions 3 mL each collected where the two degradation products (FDP 1) and (FDP 2) were isolated in pure form at polarity (9.5: 0.5) methylene chloride/methanol of the mobile phase. The two degradants were obtained in a pure form as solids after being collected separately, concentrated and the mobile phase removed (Ahmed et al. 2020).

## Cell culture

Normal Human Skin Fibroblasts (NHSF) cell line was obtained from Nawah Scientific Inc., (Mokatam, Cairo, Egypt). Cells were maintained in DMEM media supplemented with 100 mg/mL of streptomycin, 100 units per mL of penicillin, and 10% of heat-inactivated fetal bovine serum in humidified, 5% (v/v) CO_2_ atmosphere at 37 °C.

## Cytotoxicity assay

Cell viability was assessed by SRB assay. Aliquots of 100 ml cell suspension (5 × 10^3^ cells) were in 96-well plates and incubated in complete media for 24 h. Cells were treated with another aliquot of 100 mL media containing (FVP, FDP 1, FDP 2, and doxorubicin as positive control) at various concentrations ranging from (0.01, 0.1, 1, 10, 100 mm). After 72 h of drug exposure, cells were fixed by replacing media with 150 mL of 10% TCA and incubated at 4 1C for 1 h. The TCA solution was removed, and the cells were washed 5 times with distilled water. Aliquots of 70 mL SRB solution (0.4% w/v) were added and incubated in a dark place at room temperature for 10 min. Plates were washed 3 times with 1% acetic acid and allowed to air-dry overnight. Then, 150 mL of TRIS (10 mM) was added to dissolve the protein-bound SRB stain; the absorbance was measured at 540 nm using a microplate reader. The percentage of cell viability was calculated by using the following formula (Basiouni et al. 2020):

$$ \text{\% viability =} \; \frac{\text{A of treated cells}}{\text{A of untreated cells}}  {\text{ X 100}}$$

## Results and discussion

### HPLC method development and optimization

The chromatographic technique was created by choosing a mobile phase and a stationary phase that were both adequate for separating FVP from its degradation products. To determine chromatographic conditions ideally suited to the structural properties of FVP, preliminary tests were undertaken using a C18 column (150 × 4.6 mm, 5 mm) and attempting several mobile phases with varied polarity. Water as the aqueous phase was not suited since the peak shape and symmetry were unsatisfactory, thus a mobile phase with a high proportion of an organic modifier was used. The form and symmetry of the peaks were then improved by screening potassium dihydrogen phosphate buffers at various concentrations (10–50 mM) and pH values (3–5).

**Fig. 1:**
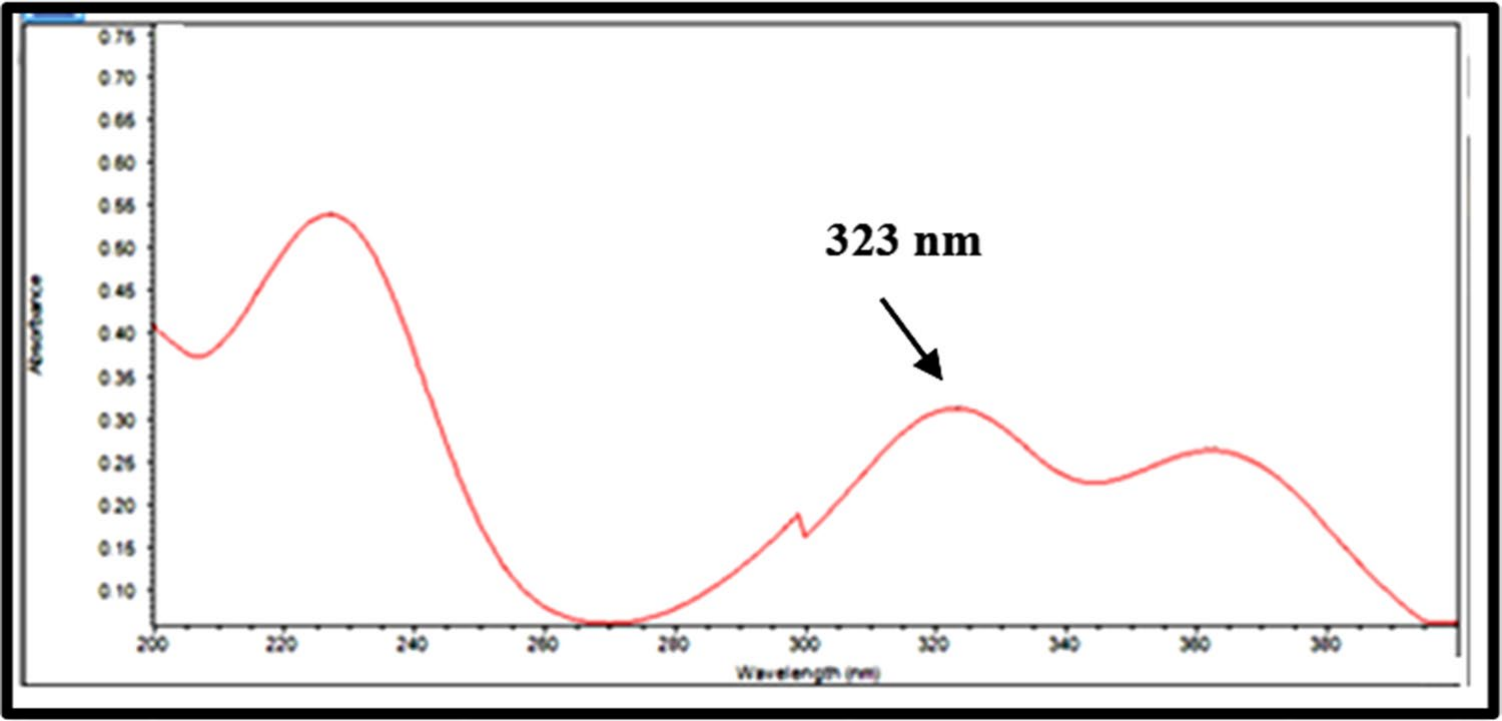
UV Spectrum of Favipiravir

**Fig. 2:**
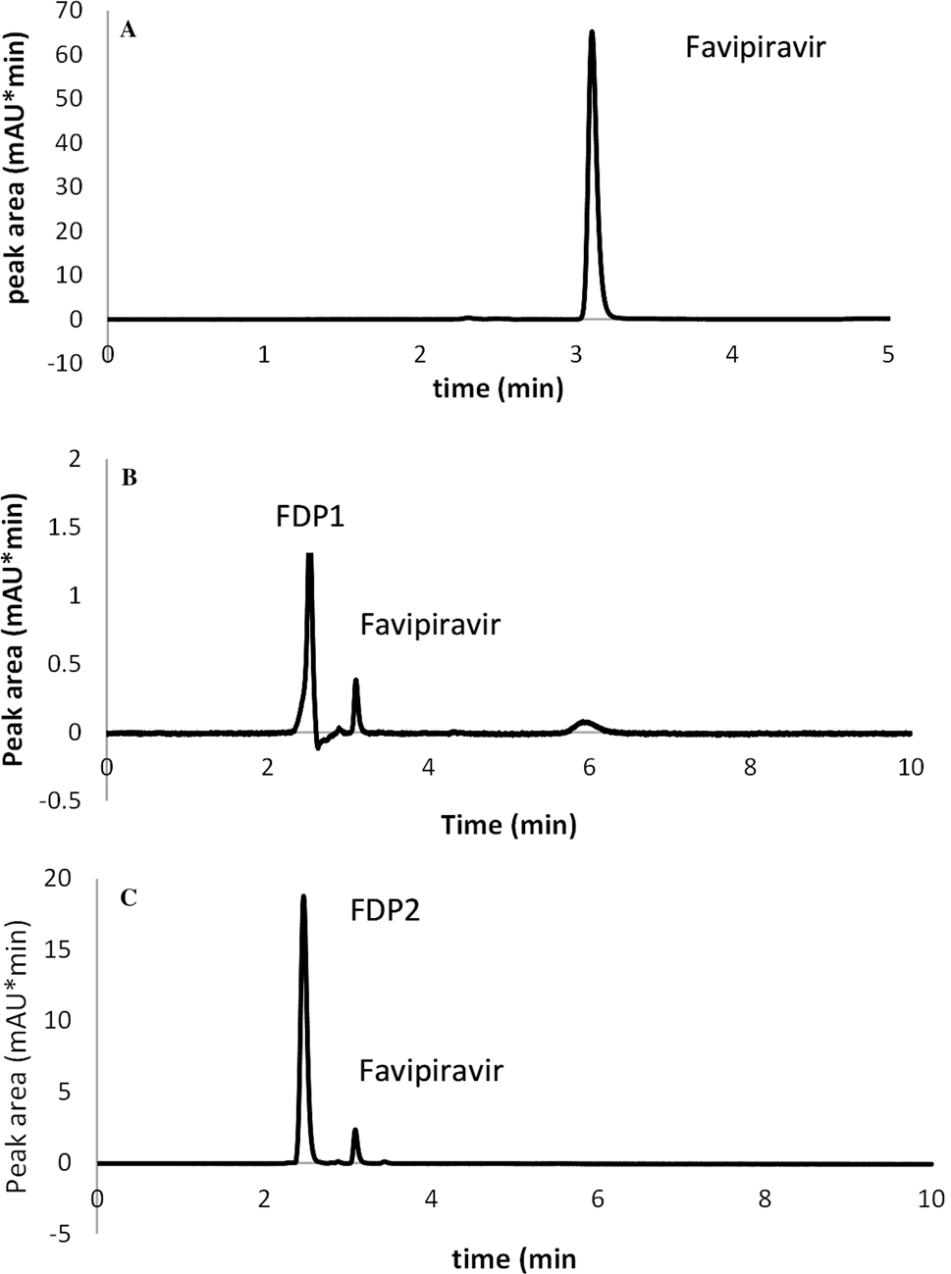
HPLC Chromatograms of (**A**) Favipiravir (10 µg/mL), (**B**) oxidative degradation (FDP1) and (**C**) alkaline degradation (FDP2)

Peak tailing was seen when using low concentrated phosphate buffers, however, the pH 2.5 buffer worked well since the pH was significantly lower than the pKa of FVP (pKa = 5.1). Following UV scanning of the drug solution, FVP was discovered at 332 nm, which was reported as the wavelength of maximum absorbance (max) (Fig. 1). Acceptable peak shapes and resolution between FVP and its degradation products were observed using isocratic elution as described under chromatographic conditions, and the chromatograms shown in Fig. 2 were acquired using the optimized conditions for FVP separation from its alkaline and oxidative degradation products. In the presence of FVP degradation products, Table 1 illustrates the results of system suitability parameters for FVP.

**Table 1:** System suitability parameters for Favipiravir in presence of its oxidative and alkaline degradates

Parameter	FDP (1)	FDP (2)
Resolution	1.41	1.77
Selectivity	1.86	1.97
No. of theoretical plates	16,056	16,040
Capacity factor	3.60	3.58
Symmetry	1.34	1.32

## Forced degradation study

According to ICH regulations, FVP 's degradation behavior was investigated, and the drug was discovered to be unstable under oxidative and alkaline circumstances.

All degradation samples were neutralized, as in alkaline hydrolysis, or left to bubble off the remaining hydrogen peroxide. Before HPLC injections, all samples were diluted with the mobile phase. All these steps demonstrate that the degradation products isolated on the HPLC chromatograms were degradants rather than residual reagent peaks. Furthermore, the existence of degradation products in the sample was confirmed by using Flash Chromatography followed by TLC to isolate them as single compounds.

When FVP was subjected to oxidative degradation, one degradation product (FDP 1) was detected using HPLC – UV. Under alkaline hydrolysis, one degradation product (FDP2) was also produced.

## Method validation

### Linearity

From the standard FVP solution, solutions at six different concentrations were generated, and a calibration curve was built by graphing the peak regions as a function of concentration. As indicated in Table 2, the regression equation was *Y* = 0.4434 X + 0.153, and the correlation coefficient was 0.9994, suggesting that the suggested approach is linear.

**Table 2:** Summary of validation parameters of the chromatographic method used for determination of Favipiravir

Parameter	Favipiravir
*Linearity*	
Regression equation	*Y* = 0.4434 X + 0.153
Range (µg/mL)	0.5 – 100
Correlation coefficient (r)	0.9994
Slope	0.4434
Intercept	0.153
LOD(µg/mL)	0.037
LOQ (µg/mL)	0.122
*Precision*	
Repeatability (Intra-day) (% RSD) *	
QCL (2 µg/mL)	1.36%
QCM (20 µg/mL)	0.93%
QCH (80 µg/mL)	1.64%
Intermediate precision (Inter-day)	
(% RSD) *	
QCL (2 µg/mL)	0.89%
QCM (20 µg/mL)	1.64%
QCH (80 µg/mL)	1.58%
*Accuracy*	
(Mean ± S.D) **	
QCL (2 µg/mL)	99.05 ± 0.58
QCM (20 µg/mL)	101.21 ± 0.14
QCH (80 µg/mL)	98.20 ± 0.55

^*^RSD: relative standard deviation

^**^Expressed mean of three replicates

## Precision

By assessing solutions of three distinct concentrations on the same day and three consecutive days, the repeatability (intra-day) and intermediate precision (inter-day) were determined. Relative standard deviations were under 2%. (Table 2). As a result, the procedure was deemed accurate enough.

## Accuracy

Spiking FVP into synthetic solutions with degradation products at three different concentrations (2, 20, and 80 µg/mL) in triplicate were used to test the method's accuracy. Then, for each of these three FVP concentrations, compute the % recoveries (Table 2). Recoveries of a reasonable percentage were achieved (between 98 and 101 percent). As a result, the established approach for detecting stability is precise.

## Specificity

Three laboratory-prepared mixes of the oxidative and alkaline degradation products **(FDP1 & FDP2)** with known amounts of the intact medication inside the linear area were analyzed to determine selectivity. The degradation products and intact FVP did not interact with each other.

## Detection limit (LOD) & quantitation limit (LOQ)

Table 2 shows that the LOD and LOQ, respectively, are 0.037 and 0.122 g/mL.

## Robustness

**Fig. 3:**
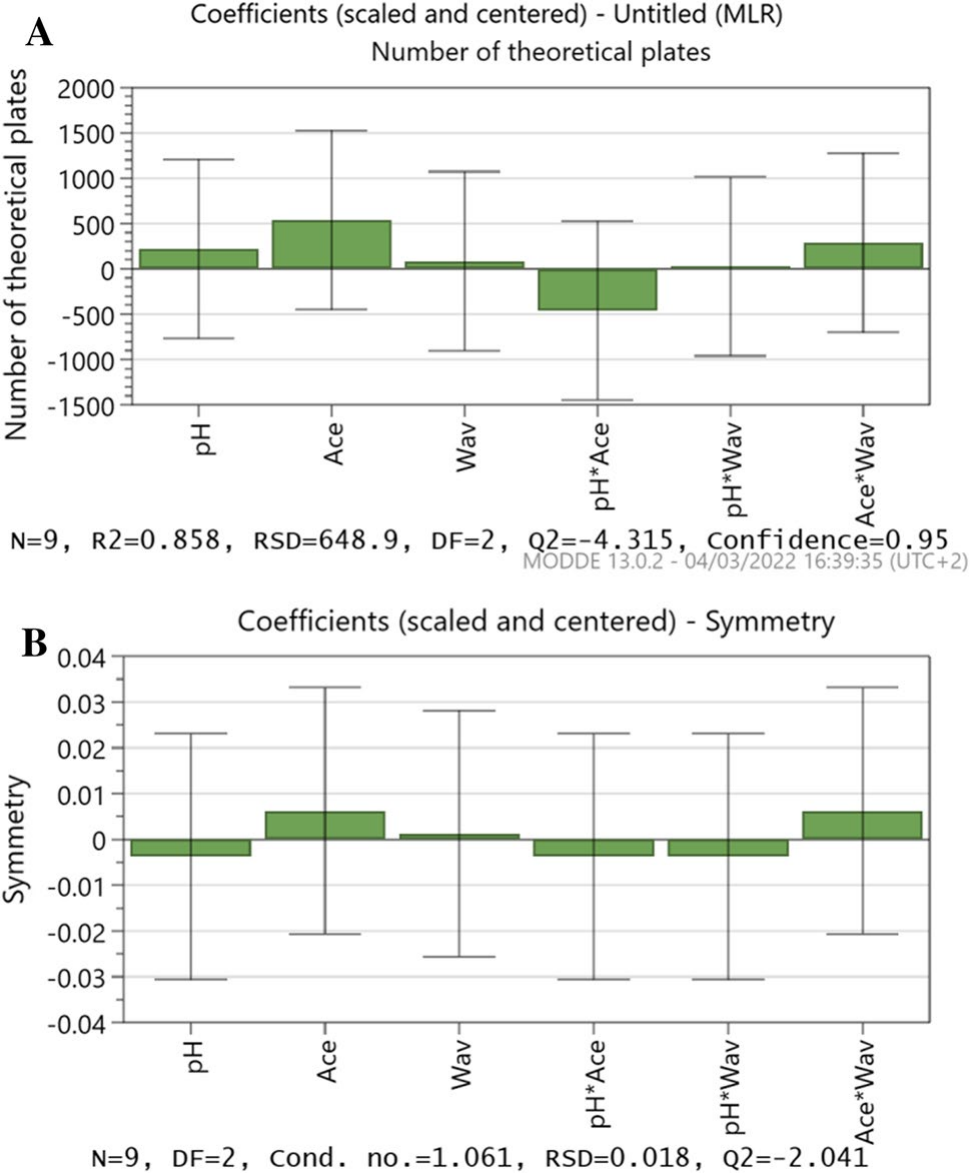
Coefficients plots for method robustness (**A**) Number of theoretical plates, and (**B**) Symmetry

The robustness of the approach was evaluated using Plackett-design. Burman's Small variations from the procedure conditions were investigated, and the resulting reactions were noted. Table 3 shows the results of eleven randomized runs with three center points. At *p* values less than 0.5, all parameters were determined to be non-significant, and the coefficient plot (Fig. 3) verified the non-significance of these factors in terms of theoretical plates and FVP selectivity.

**Table 3:** Design of experiment (DOE) for Favipiravir robustness testing

Exp. No	pH	Acetonitrile	Wavelength
1	2.7	48	321
2	2.7	52	321
3	2.7	52	325
4	2.3	52	325
5	2.7	48	325
6	2.3	52	321
7	2.3	48	325
8	2.3	48	321
9	2.5	50	323
10	2.5	50	323
11	2.5	50	323

Characterization of degradation products

### Characterization of degradation products

**Fig. 4:**
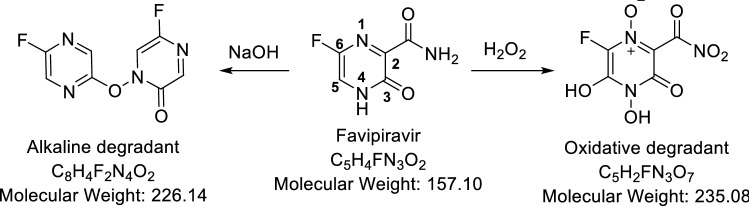
Favipiravir, its oxidative and alkaline degradants

At the inception, intact FVP has been confirmed by 1H-NMR and Mass spectroscopy. In the 1H-NMR spectrum, four protons have been detected at δH 13.37 (singlet for 1H), 8.72 (singlet for 1H), and 8.50 (doublet for 2Hs) ppm of cyclic NH, aromatic H, and NH2 respectively. Besides, ESI–MS has revealed the protonated molecule at 158 m/z in the positive mode and 156 m/z in the negative mode which was attributed to [M + 1]^+^ and [M-1]^+^ of FVP respectively (Fig. 4).

In the alkaline conditions, FVP was converted to the depicted structure in (Fig. 4) firstly, the acidic proton was abstracted by alkali to afford the charged phenoxide structure (I) followed by the alkaline hydrolysis of the amide to the corresponding acid (II) as reported (Lopez et al. 2003). The afforded acid was then decarboxylated under the effect of the alkali and heating (Diefallah, 1979; Mohammed Farrag El-Behairy and Sundby, 2013) to afford compound III. Then, compound III attached its resonance structure to afford the alkaline degradant IV. Compound IV (alkaline degradant) showed 227 m/z in ESI–MS analysis that corresponding to molecular ion peak [M + 1]^+^. The ^1^H-NMR spectrum revealed the presence of four singlet signals (each for 1H) that were noticeable in the aromatic region at *δ*_H_ 8.30, 7.55, 7.42, and 7.29 ppm.

**Fig. 5:**
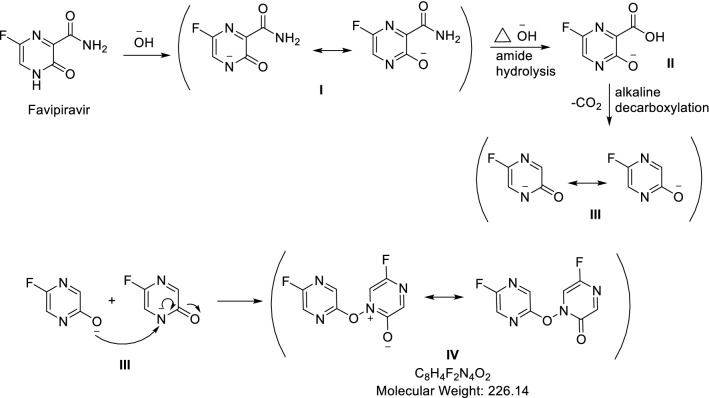
Postulated mechanism of formation of alkaline degradant

**Fig. 6:**
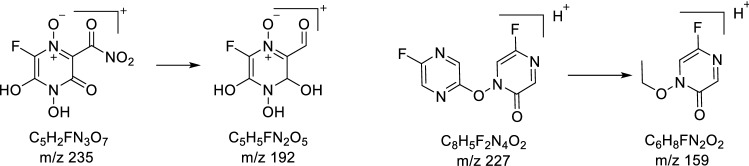
Fragmentation pattern of both oxidative and alkaline degradants

**Fig. 7:**
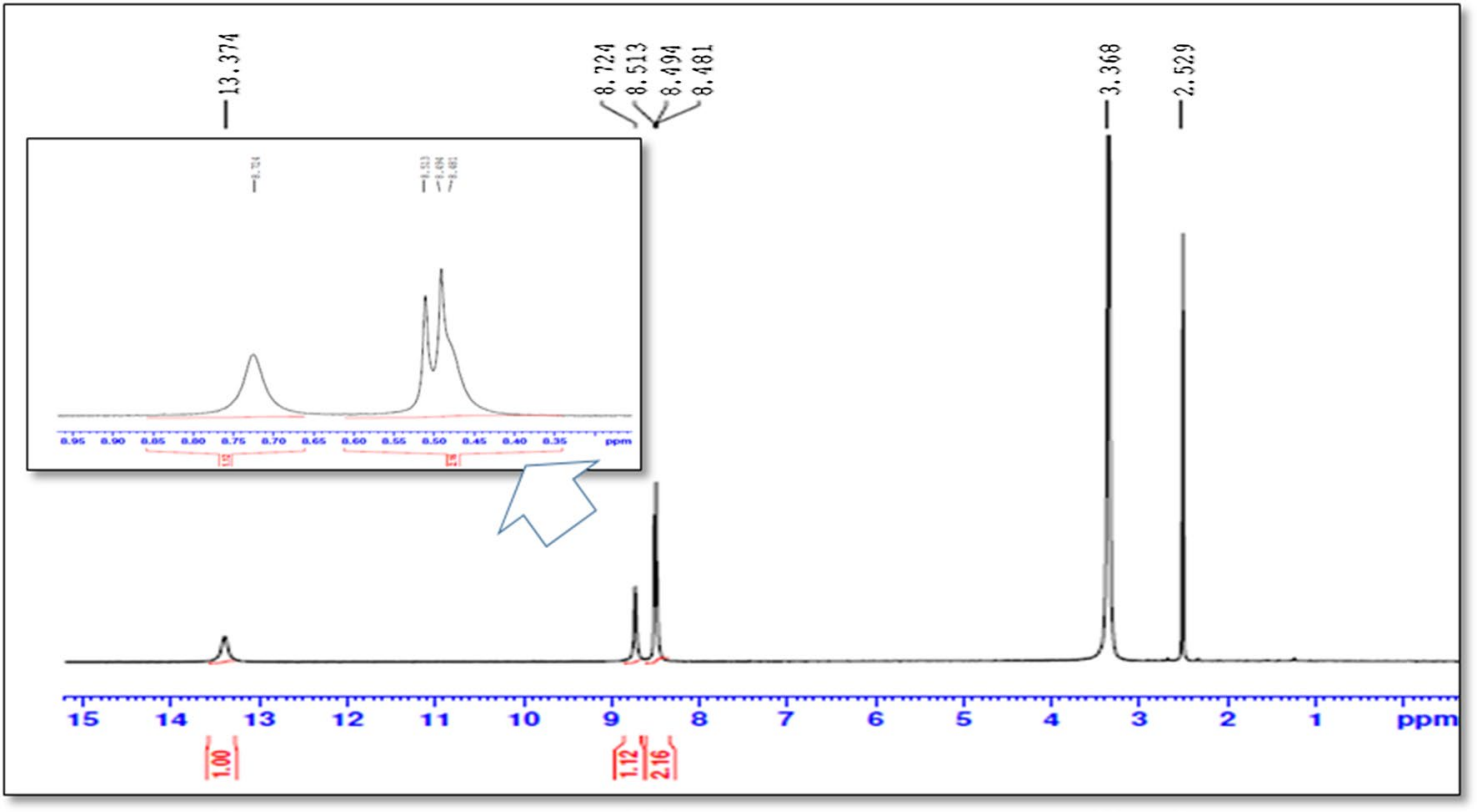
^1^H-NMR spectrum of intact Favipiravir (400 MHz, DMSO-*d*_*6*_)

**Fig. 8:**
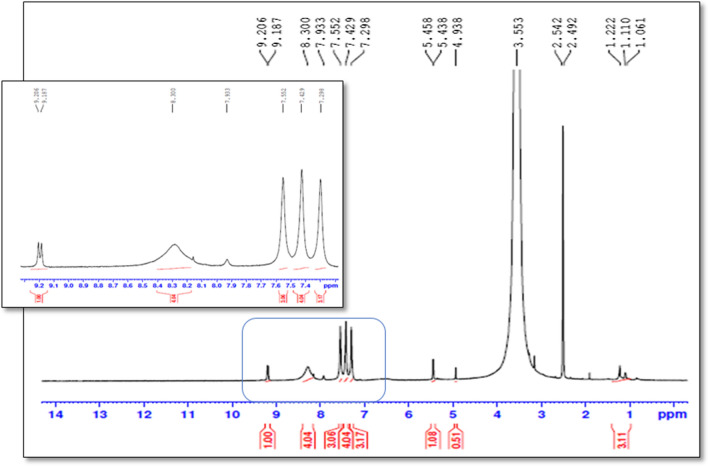
^1^H-NMR spectrum of the alkaline product (FDP 2) (400 MHz, DMSO-*d*_*6*_)

**Fig. 9:**
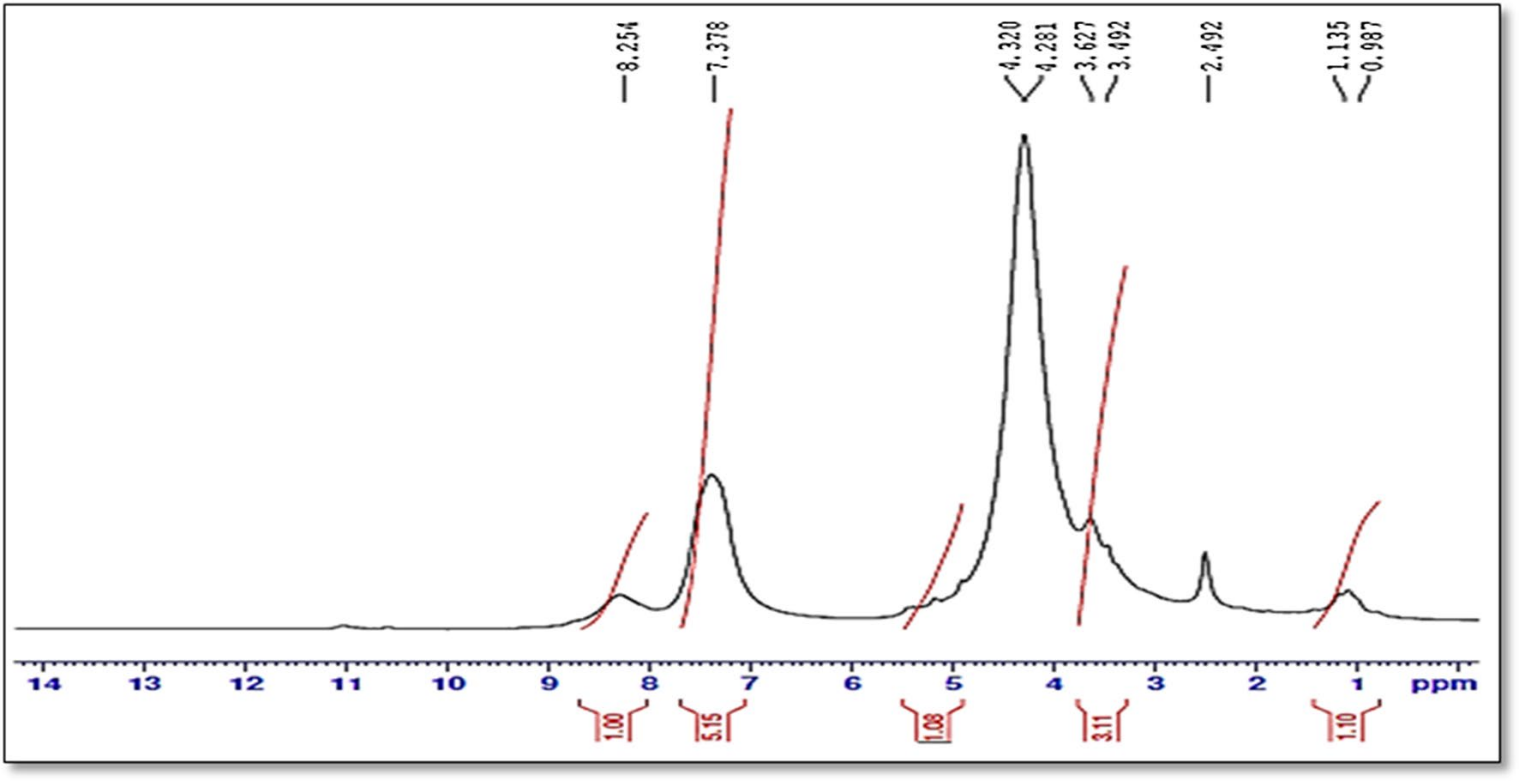
^1^H-NMR spectrum of the oxidative product (FDP 1) (400 MHz, DMSO-*d*_*6*_)

**Fig. 10:**
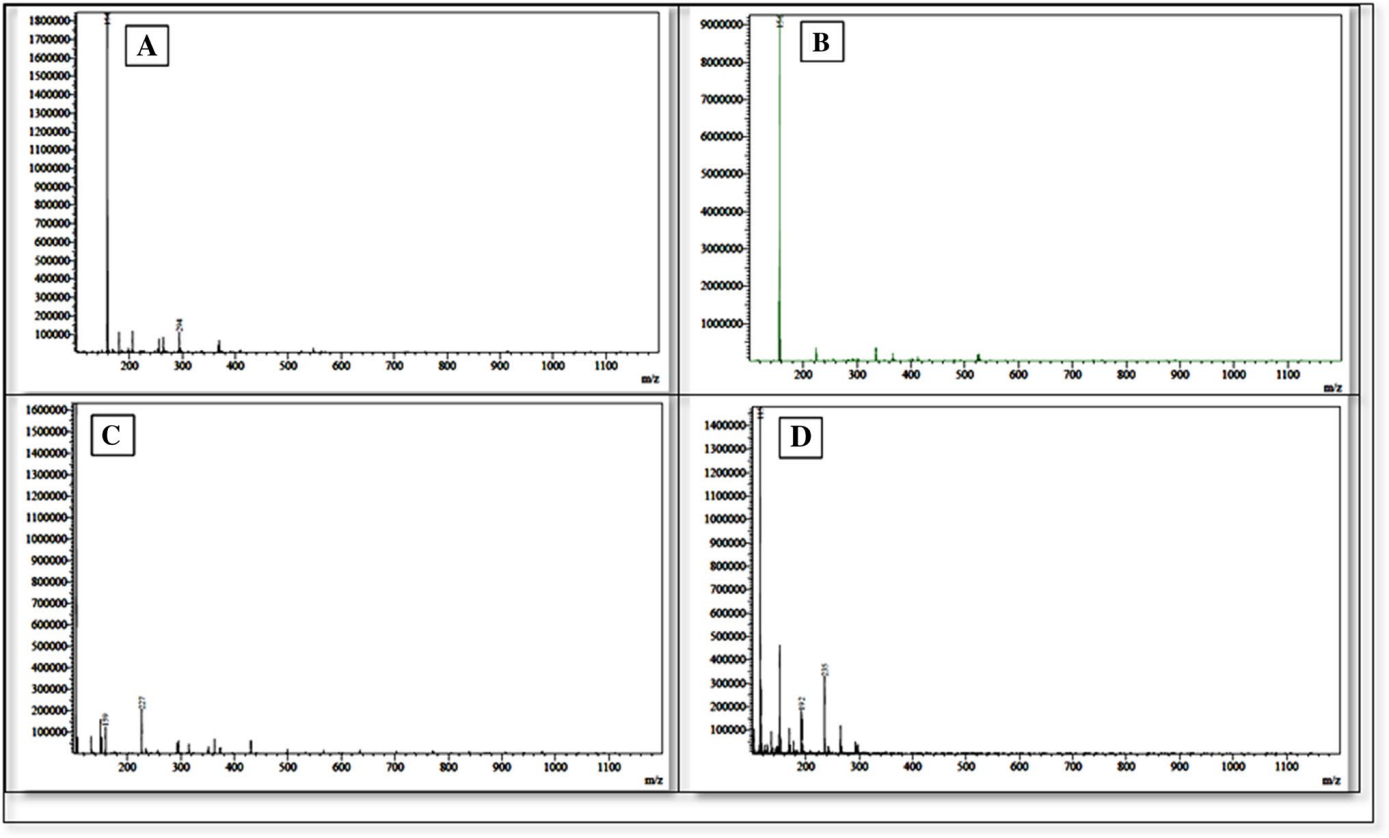
MS Spectrum of intact Favpiravir in the positive (**A**) and negative (**B**) modes, alkaline (**C**), and oxidative (**D**) products

For the oxidative degradation, as depicted in (Fig. 5), all protons, at positions 4, and 5 of FVP have been oxidized to OH. While NH_2_ of carboxamide at position 2 converted to NO_2_. In addition, N-oxide has been formed from nitrogen at position 1. The postulated structure has been confirmed by ^1^H-NMR and Mass spectroscopy (Figs. 6, 7, 8, 9 and 10). In the ^1^H-NMR of the oxidative degradant, all characteristic signals of FVP have disappeared while two broad singlet signals were detected at *δ*_H_ 8.25, 7.37 ppm that are corresponding to both OHs at position 4 and 5. In ESI–MS, a molecular ion peak at 235 m/z was observed that represents [M]^+^.

## Cytotoxicity assay

**Fig. 11:**
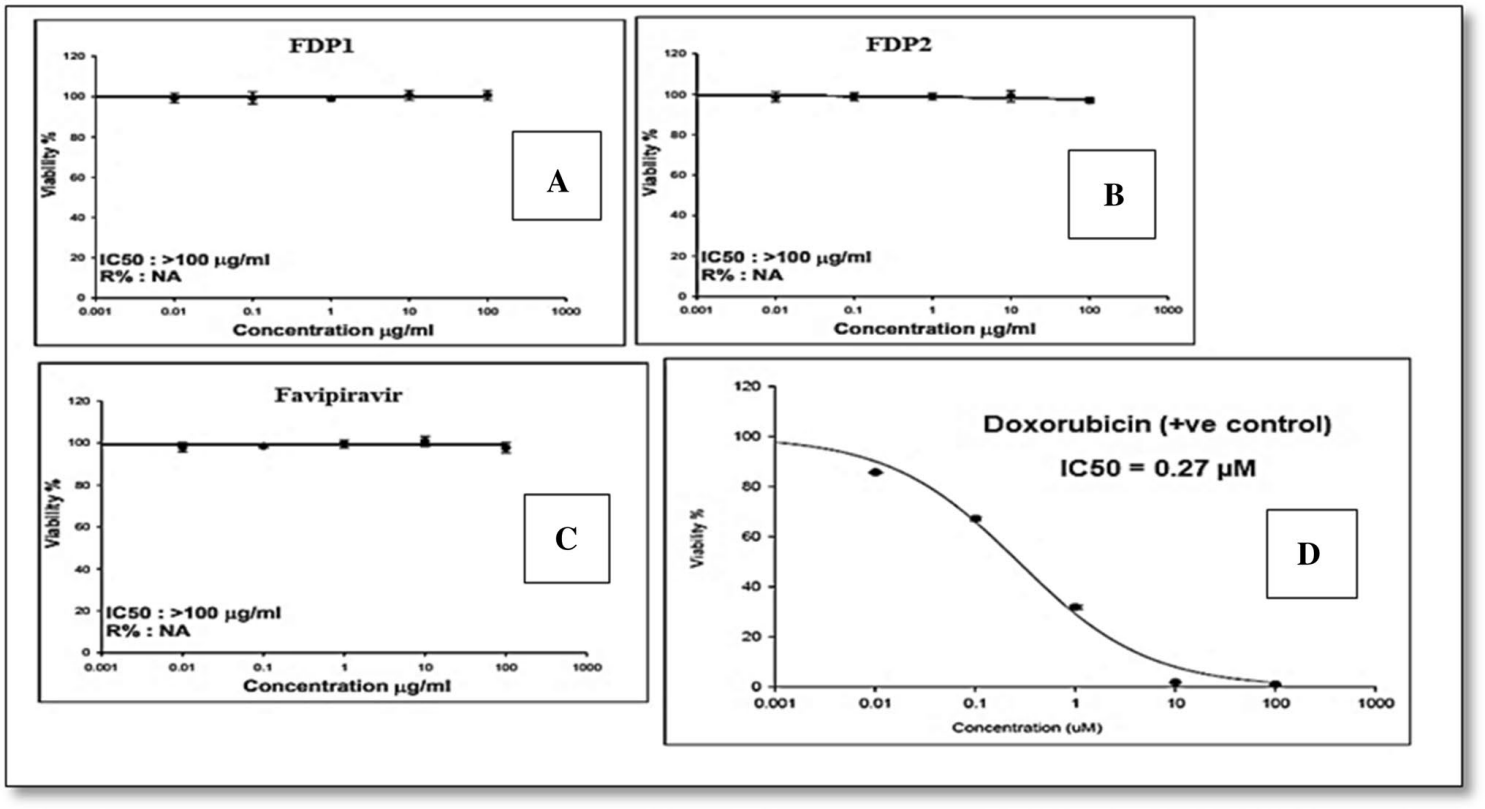
In vitro Cytotoxicity of (**A**) FDP1; **B** FDP2; **C** Favipiravir, and **D** Doxorubicin in increasing concentrations ( 0.01 – 100 uM) incubated in Human Skin Fibroblast cell lines (NHSF) using SRB viability assay. Data points are expressed as mean ± SD (*n* = 3)

**Fig. 12:**
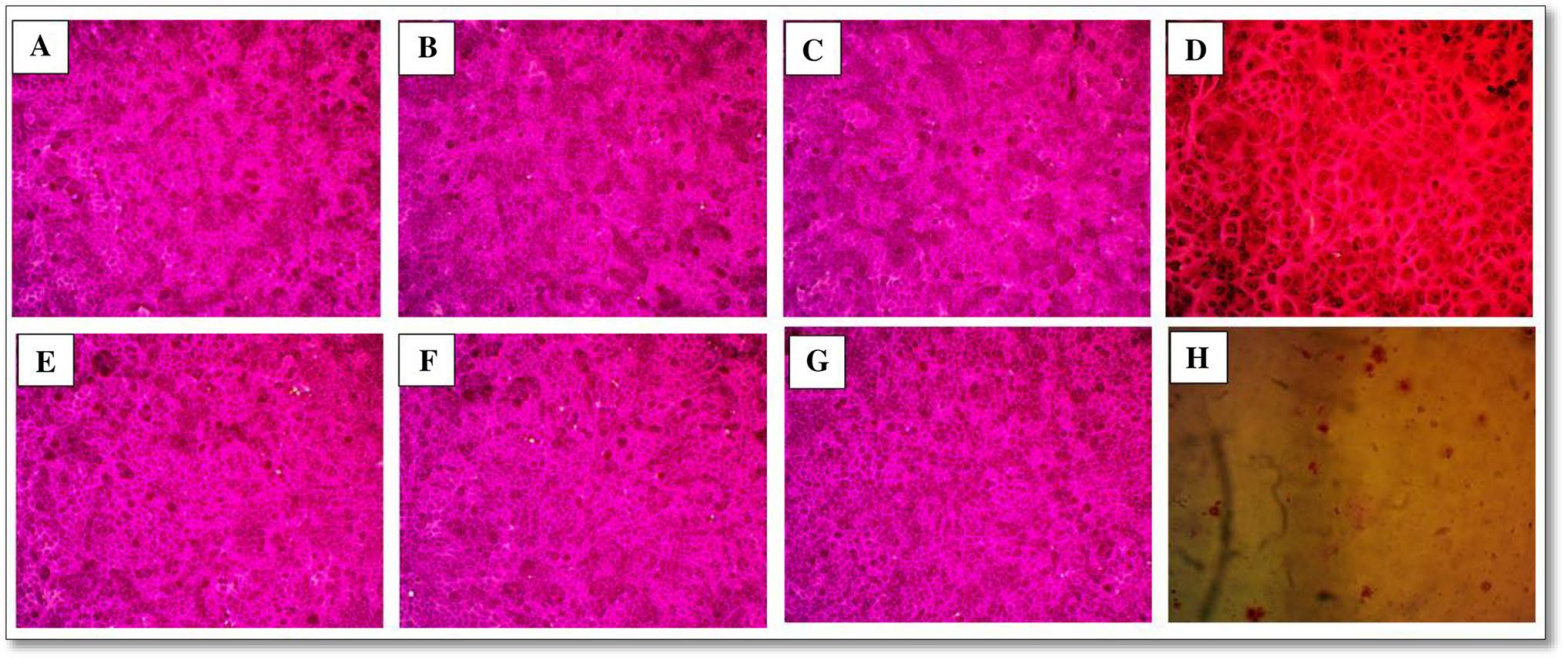
Optical microscope-stained images of cytotoxicity assays at NHSF cell line **A** FDP1 (0.1 μM), **B** FDP 2 (0.1 μM), **C** Favipiravir (0.1 μM), **D**: Doxorobucin (0.1 μM), **E** FDP 1 (100 μM), **F** FDP 2 (100 μM) **G** Favipiravir (100 μM) and **H** Doxorubicin (100 μM), Magnification power: 200x

Sulforhodamine B (SRB) cytotoxicity testing is one of the most often used in vitro procedures for predicting a substance's potential toxicity in cell culture. In cell-based investigations, SRB is a sensitive and repeatable test for cytotoxicity. In slightly acidic circumstances, SRB binds stoichiometrically to protein components of cells fixed to tissue culture plates. The dye is then removed from stained cells and quantified using a colorimetric assay in basic conditions. The colorimetric analysis yields a total protein mass estimate that is proportional to the number of cells present. FVP and its breakdown products (FDP 1 and FDP 2) were tested in vitro against NHSF cell lines to determine their potential toxicity and/or safety (Ahmed et al. 2020; Mohammed F. El-Behairy et al. 2021). The effect of these chemicals on the viability and proliferation of the NHSF cell line was assessed using the SRB assay, as well as photos taken with an optical microscope to detect any morphological changes on the examined cells. After 72 h of incubation, the percentage of cell viability was assessed at five different doses (0.01. 0.1, 1, 10, and 100 µg/ml) in comparison to doxorubicin, a typical cytotoxic agent. For the oxidative and alkaline degradation products (FDP1 & FDP2), cell viability reached 100% at concentrations of 0.01 mM. Favipiravir, FDP 1, and FDP 2 at two concentration levels (0.1 and 100 µg/ml) were compared to doxorubicin at the same concentration levels, as shown in (Fig. 11). It clearly reveals that at 0.1 and 100 mM of FVP, FDP 1, and FDP 2 (IC_50_ = ˃ 100 µg/ml), no morphological alterations occur (Fig. 12). This demonstrates that FVP and its breakdown products are non-toxic at concentrations more than 100 µg/ml.

## Conclusion

Favipiravir, its oxidative and alkaline degradation products (FDP1 & FDP2) were separated using HPLC – UV on a reversed-phase Eclipse XDB C18 column (4.6 150 mm, 5 mm) using isocratic mode with a flow rate of 1 mL/min and detected at 332 nm. The method was validated according to the ICH guidelines and was linear over the concentration range of 0.5 to 100 µg/mL. In vitro evaluation of FVP and the produced degradants showed no cytotoxicity effect on normal skin fibroblasts cell lines with IC_50_ ˃100 µg/ml with no signs of toxicity upon examination of the treated NHSF cells under the optical microscope.

## Abbreviations

DOE: Design of experiment
FVP: Favipiravir
FDP1: Oxidative degradation product
FDP2: Alkaline oxidative product
HPLC: High-performance liquid chromatography
ICH: International conference on harmonization
LOD: Detection limit
LOQ: Quantitation limit
NHSF: Normal skin fibroblasts cell lines
TLC: Thin layer chromatography.
